# Quantitative analysis of pathological findings identified clinical heterogeneity in nonspecific interstitial pneumonia with organising pneumonia overlap

**DOI:** 10.1038/s41598-025-04259-y

**Published:** 2025-06-03

**Authors:** Masato Asaoka, Hideya Kitamura, Tae Iwasawa, Koji Okudela, Tamiko Takemura, Takashi Ogura

**Affiliations:** 1 Internal Medicine, Keiyu Hospital, 3-7-3 Minato-Mirai, Nishi-ku, Yokohama-shi, Kanagawa, Japan; 2 Department of Respiratory Medicine, Kanagawa Cardiovascular and Respiratory Center, Kanagawa, Japan; 3 Department of Radiology, Kanagawa Cardiovascular and Respiratory Center, Kanagawa, Japan; 4 Department of Pathology, School of Medicine, Yokohama City University, Kanagawa, Japan; 5 Department of Pathology, Kanagawa Cardiovascular and Respiratory Center, Kanagawa, Japan

**Keywords:** Autoimmune disease, Histology, Interstitial lung disease, Lung fibrosis, Radiology and other imaging, Respiratory tract diseases, Diseases, Medical research

## Abstract

The pathological classification of nonspecific interstitial pneumonia (NSIP) with organizing pneumonia (OP) overlap (NSIP/OP overlap) remains complex due to overlapping pathological features, and its heterogeneity is not well understood. We retrospectively analysed adult patients with interstitial lung disease (ILD) diagnosed with NSIP/OP overlap via surgical lung biopsy. Patients were pathologically subclustered using an unbiased clustering method, and clinical, radiological, and prognostic differences were examined. Among 38 patients, two pathological clusters were identified: Cluster 1, characterized by fibrotic changes with mild inflammation, and Cluster 2, exhibiting intense inflammation with fibrosis. While both clusters initially responded well to treatment, Cluster 2 demonstrated progressive ILD deterioration and a higher frequency of pulmonary fibrosis. Cluster 2 was also associated with hypoxia, reduced pulmonary function, elevated erythrocyte sedimentation rate, and greater consolidation on chest computed tomography. Based on these findings, we have identified NSIP/OP overlap is a heterogeneous and progressive disease and pathological findings at diagnosis significantly influence both initial ILD severity and long-term prognosis. Our findings highlight the need for tailored long-term management strategies based on early histopathological evaluation.

## Introduction

The pathological overlap between nonspecific interstitial pneumonia (NSIP) with organising pneumonia (OP) (NSIP/OP overlap) is closely associated with connective tissue disease (CTD), particularly polymyositis/dermatomyositis^1–8^. NSIP/OP overlap is also present in patients with idiopathic interstitial pneumonia (IIP), including those who are anti-aminoacyl-tRNA synthetase (ARS) antibody-positive^9–11^. While NSIP itself can contain OP involvement in less than 20%^12^, IIP with NSIP/OP overlap is considered unclassifiable due to overlap of histologic patterns based on the American Thoracic Society/European Respiratory Society classification statement^13^. Nevertheless, NSIP/OP overlap is one of the criteria for interstitial pneumonia with autoimmune features and could be regarded as a distinctive morphological and pathological pattern^14^. The NSIP/OP pattern has been reported as the second most frequently observed radiological pattern in patients with myositis-associated interstitial lung disease (ILD) and ILD positive for myositis-specific autoantibodies, following NSIP^8,9^.

Most previous studies have described the clinical characteristics of NSIP/OP overlap by comparing it with other distinct pathological patterns, such as usual interstitial pneumonia (UIP), NSIP or OP^15–17^. One study indicated that NSIP/OP overlap had a good survival rate, while its clinical outcome was worse than OP^15^. These analyses greatly contributed to our understanding of the general features of NSIP/OP overlap; however, they could not address the individual variation in NSIP/OP overlap, which causes diverse clinical outcomes. In addition, the relationship between the clinical, radiological, and pathological diversity of NSIP/OP overlap and the clinical outcomes, including treatment response, has remained unexplored. Furthermore, a key limitation in the analysis is the absence of a clearly established definition for diagnosing NSIP/OP based on radiological findings.

Here, we retrospectively evaluated patients with ILD who were pathologically diagnosed with NSIP/OP overlap based on surgical lung biopsy (SLB) and investigated the variations in pathological components to identify the association between the condition’s pathological heterogeneity and patient clinical courses.

## Methods

### Patient selection and study design

Of 3864 patients with ILD who first visited our centre between April 2012 and December 2018, 506 patients underwent diagnostic SLB. Among them, 50 patients were initially diagnosed with a NSIP/OP overlap pattern via SLB. The diagnosis was confirmed through a multidisciplinary discussion (MDD) involving at least three pulmonologists, two radiologists, and two pathologists. Six were excluded due to transfer to other hospitals before the MDD diagnosis. A pathological extent of more than 20% OP was required for the diagnosis of NSIP/OP overlap. Six additional cases were excluded post-MDD: three were unclassifiable (with overlapping features of UIP and NSIP), two were reclassified as fibrotic NSIP, and one as OP. Ultimately, 38 patients were included in this study.

This study was approved by the Institutional Review Board of Kanagawa Cardiovascular and Respiratory Center (Approval Number: KCRC-19-0008). In accordance with ethical guidelines, the purpose and conduct of the research were disclosed to all participants, ensuring they had the opportunity to refuse participation or withdraw. Given the retrospective nature of the study, which relied solely on previously obtained data and samples without new invasions or interventions, the Institutional Review Board of Kanagawa Cardiovascular and Respiratory Center waived the requirement for written informed consent. All methods in this study were conducted in compliance with relevant guidelines and regulations, including the Declaration of Helsinki.

Clinical data were retrospectively collected from medical records, and pulmonary function tests performed during a minimum three-year follow-up period were reviewed. Anti-ARS antibodies were detected using RNA and protein immunoprecipitation assays.

High-resolution computed tomography (HRCT) images with 1.0 mm-thick sections taken during a single breath-hold were collected every 12 ± 3 months when available. Axial distribution was classified as peripheral, peribronchovascular, or diffuse. Overall zonal predominance in the upper lung, lower lung, or random distribution was also evaluated for each patient. The presence of HRCT findings, including ground-glass opacity (GGO), consolidation, reticulation, traction bronchiectasis, subpleural curvilinear shadow, cyst, and honeycomb, was identified based on the Glossary of Terms by the Fleischner Society^18^. Mediastinal findings, including the ratio of the pulmonary artery to the ascending aorta, pleural effusion, and lymph node adenopathy, were also reviewed. Finally, the overall HRCT pattern of each case was diagnosed as either “UIP,” “probable UIP,” “indeterminate for UIP,” or “alternative diagnosis” by at least two well-trained radiologists based on the 2022 guidelines for idiopathic pulmonary fibrosis^19^. Discordance in the diagnosis between the two radiologists was assessed by a third radiologist, and the final diagnosis was made through discussion among the three radiologists. To analyse the HRCT images quantitatively, we utilised a deep learning-based ILD analysis system^20^. The extent of the following eight features—normal, emphysema, GGO, consolidation, consolidation with fibrosis, reticulation, honeycomb, and traction bronchiectasis—was calculated for all images obtained from the participants at their first visit. The changes in each HRCT finding were graded as resolved, improved, unchanged or worsened on every follow-up CT scan, if available.

Lung tissue samples were collected from at least one area of both the upper and lower lung lobes. All specimens were stained with haematoxylin–eosin (HE) and Elastica van Gieson (EVG) and evaluated according to previous studies^21–24^. Diagnosis and evaluation were performed by at least two well-trained pathologists with > 20 years of experience in diagnosing ILDs. Disagreements between the two pathologists were resolved through discussion until a consensus was reached. NSIP/OP overlap was defined as the absence of a UIP pattern, widespread OP foci with preserved background lung architecture, and the presence of diffuse interstitial inflammation and/or fibrosis^12,13,25,26^.

### Dimension reduction and clustering

For further analysis, 13 additional pathohistological features were evaluated in each specimen resected from the lower lobes of the participants’ lungs and scored using a four-point scale (absent, mild, moderate, and severe): alveolar epithelial injury, bronchiolitis, interstitial cellular infiltration, collapse, fibrosis, lymphoid follicle, mural incorporation fibrosis, OP, plasma cell infiltration, pleural fibrosis, pleuritis, traction bronchiectasis, and type II pneumocyte hyperplasia.

Principal component analysis (PCA), an unsupervised pattern recognition technique, was used to integrate the 13 pathohistological parameters and visualise the similarities and differences between the patients’ pathohistological characteristics. Original pathohistological data, including the 13 features, were projected onto two dimensions using PCA, and all patients were subclustered histologically based on the K-means algorithm. All PCA and clustering processes were performed using the Python software. Details are provided in the “Statistical analysis” section below.

### Statistical analyses

Missing data were removed via listwise deletion. Data were analysed using the Python software, version 3.8.12 (Python Software Foundation, Delaware, United States), with statistical libraries NumPy 1.20.1, pandas 1.2.4, scikit-learn 0.24.1, and SciPy 1.6.3.

All data are presented as means ± standard deviations, medians with interquartile ranges, or counts with percentages. Parametric variables were compared using t-tests, and non-parametric variables were compared using the Wilcoxon–Mann–Whitney test. Categorical variables were analysed using the chi-squared test. Statistical significance was set at *P* < 0.05 significant. Receiver operating characteristic (ROC) curves were plotted after calculating the areas under the curves (AUCs). The optimal cut-off values were determined using the Youden’s index.

## Results

### Patient characteristics

The patients’ baseline characteristics were listed in Table 1. Twelve patients (31.6%) were diagnosed with clinical amyopathic ILD associated with anti-ARS antibody-positive, while eight patients (21.1%) diagnosed with DM/PM. A total of 17 patients (44.7%) were confirmed to be anti-ARS antibody-positive. Anti-EJ and anti-Jo-1 were the two most frequently isolated antibodies (29.4%), followed by anti-KS, anti-PL-7, and anti-PL-12 (11.8% each), and lastly anti-OJ antibodies (5.8%). Only one case was positive for anti-MDA5 antibodies. As an initial treatment, most patients (81.6%) received systemic corticosteroid therapy. Twenty patients (64.5%) received pulse steroid therapy (methylprednisolone 500 mg/day for 3 days), followed by oral corticosteroids (0.5–1.0 mg/kg/day) with or without immunosuppressants. The most commonly used immunosuppressant in these patients was tacrolimus, administered orally at a dose of 0.0375 mg/kg every 12 h, in 31.6% of cases, followed by cyclosporine A, administered orally at a dose of 3 mg/kg/day, in 23.7% of cases.

**Table 1:** Baseline characteristics of 38 patients diagnosed as having NSIP/OP overlap.

Number of patients	38
Age (years)	59 (49–66)
Sex (male:female)	14:24
Never smoker	15 (39.5%)
BMI	24.5 ± 4.29
Diagnosis	
ILD associated with anti-ARS antibody-positive	12 (31.6%)
IIPs	11 (29.0%)
PM/DM	8 (21.1%)
Systemic sclerosis	5 (13.2%)
MCTD	1 (2.63%)
Familial history of IP	4 (10.5%)
Symptom	
Dyspnea	30 (79.0%)
Cough	22 (57.9%)
Skin rash	17 (44.7%)
Weight loss	6 (15.8%)
Fever	6 (15.8%)
Sputum	5 (13.2%)
Pulmonary function test	
TLC, % pred	82.5 ± 15.4
FVC, % pred	79.6 ± 17.1
FEV_1_, % pred	77.1 ± 19.3
DL_CO_, % pred	68.4 ± 15.6
Blood test	
LDH (U/L)	229.5 (209.3–295.5)
KL-6 (U/mL)	1414.5 (986–2439.5)
CRP (mg/dL)	0.28 (0.11–0.76)
ESR (mm/h)	26.5 (19.8–42.3)
pO_2_ (mmHg)	78.5 (73.7–83.8)
Initial treatment	
Pulse steroid therapy	20 (52.6%)
OCS + immunosuppressive	8 (21.1%)
OCS	3 (8.11%)
Anti-fibrotic therapy	2 (5.26%)
Observation	5 (13.2%)
Follow-up period (days)	1399 ± 694

*BMI* body mass index, *DL*_*CO*_ diffusing capacity of the lungs for carbon monoxide, *ESR* erythrocyte sedimentation rate, *FEV*_*1*_ forced expiratory volume in one second, *FVC* forced vital capacity, *IIP* idiopathic interstitial pneumonia, *IP* interstitial pneumonia, *KL-6* Krebs von den Lungen-6, LDH lactate dehydrogenase, *MCTD* mixed connective tissue disease, *OCS* oral corticosteroid, PM/DM polymyositis/dermatomyositis, *TLC* total lung capacity.

The radiological features of NSIP/OP overlap were assessed in this study. Almost all cases (97.4%) showed abnormal opacities in peribronchovascular distribution and lower lobe predominance patterns, and one case (2.6%) demonstrated diffuse GGO with consolidation. All patients displayed GGO, and most had consolidation (81.6%) along with reticulation (84.2%) and traction bronchiectasis (94.7%) predominantly in the bilateral lower lobes. Fifteen patients (39.5%) showed subpleural curvilinear shadow. While cysts were observed in the lungs of eight cases (21.1%), no honeycomb patterns were identified in any case. These results suggested HRCT patterns of all patients were consistent with alternative diagnosis to IPF. Mediastinal abnormalities, including pulmonary artery to aorta ratio > 1 (18.4%), pleural effusion (5.3%), and lymph node adenopathy (2.6%), were observed in some cases; however, no causal relationship with underlying diseases was identified.

### Unsupervised clustering using PCA

**Fig. 1:**
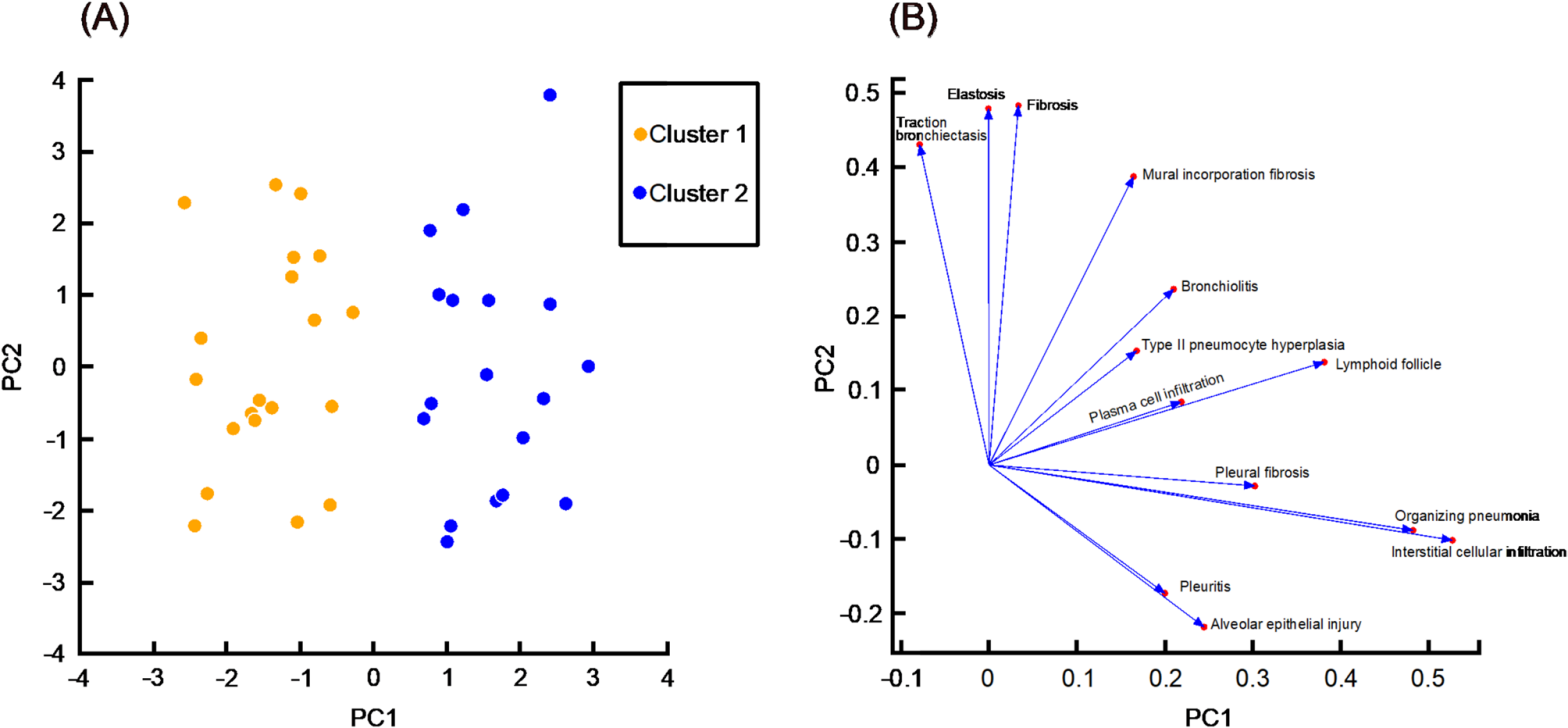
The principal component analysis based on pathological findings. The principal component analysis (PCA) score plot (**A**) showed visible relationship among 38 cases. All cases were divided into two clusters using K-means algorithm (orange, Cluster 1; blue, Cluster 2). The principal component vector plot (**B**) indicated how each pathological variable contributed to the variance in the data. PC1, first principal component; PC2, second principal component.

We performed a detailed pathological evaluation of the lower lung lobes obtained via SLB by quantitatively scoring 13 pathohistological features. All specimens showed mid to severe inflammatory cell infiltration, mural incorporation fibrosis, plasma cell infiltration, fibrosis, and type II pneumocyte hyperplasia. Most cases exhibited OP (86.8%), bronchiolitis (84.2%), elastosis (63.2%), alveolar epithelial injury (57.9%), and pleural fibrosis (50%) in the lower lobes of the lungs. In 13.2% of cases, although OP findings were not prominent in the lower lobes, extensive OP was observed in the upper lobes along with NSIP. Therefore, these cases were also considered to be consistent with NSIP/OP overlap. Lymphoid follicles with germinal centers (47.3%), and pleuritis (15.8%) were observed in several cases. Visible relationships between patients based on these 13 pathological findings were identified using PCA (Fig. 1A). Subsequently, using K-means algorithm, thirty-eight patients were divided into two clusters: 20 patients (52.6%) in Cluster 1 and 18 patients (47.4%) in Cluster 2. The principal component vector plot (Fig. 1B) indicated that OP and interstitial cellular infiltration were most strongly associated with the classification of clusters 1 and 2.

**Fig. 2:**
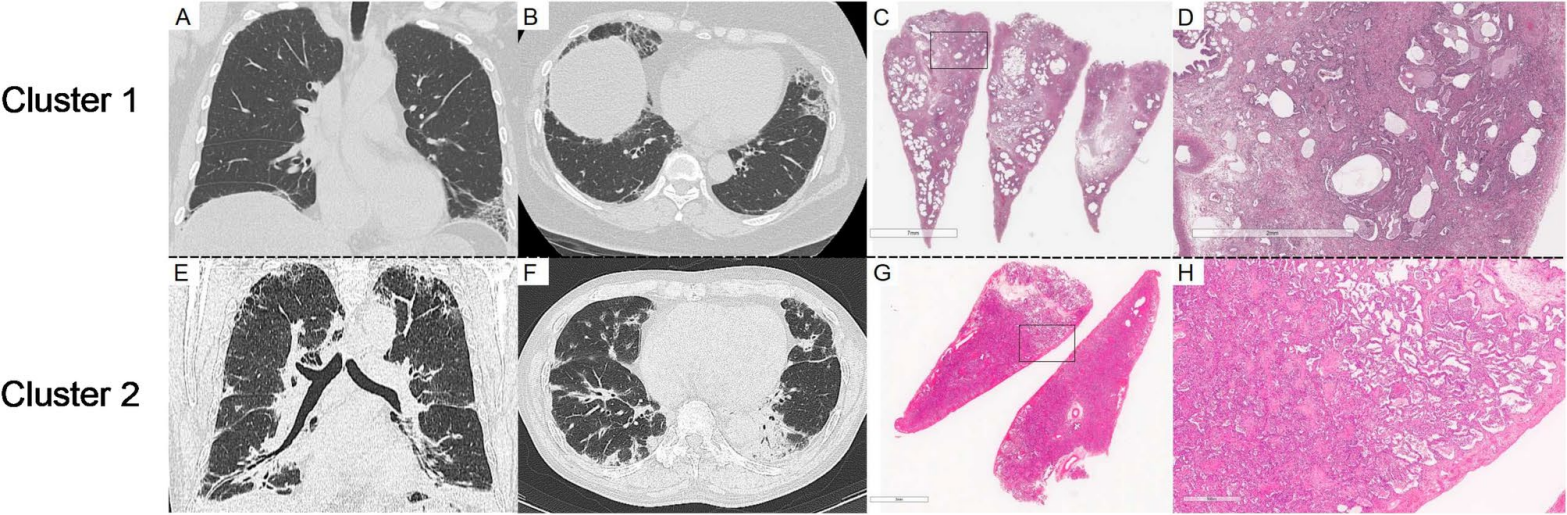
Representative radiological and pathological images of the two clusters. Chest CT findings for coronal (**A**) and axial (**B**) sections of Cluster 1, along with an overview of the haematoxylin–eosin (HE)-stained pathology specimen (**C**) and a magnified view of the region (**D**) outlined by a black square, are presented. Similarly, chest CT findings for Cluster 2 in the coronal (**E**) and axial (**F**) sections, along with an overview (**G**) and magnified view (**H**) of the lung specimens, are presented. Cluster 2 displayed a marked intensity of consolidation with volume loss in the bilateral lung fields, whereas major components of lung lesions in Cluster 1 were ground-glass opacity (GGO) and reticulation. Cluster 1 exhibited more prominent fibrosis and structural alterations in HE-stained specimens while Cluster 2 exhibited marked inflammatory exudate in the alveolar lumen.

Representative chest CT images and pathological findings for clusters 1 and 2 are shown in Fig. 2. Cluster 2 (Fig. 2E,F) demonstrated more extensive consolidation in bilateral lung fields than Cluster 1 (Fig. 2A,B). Furthermore, compared with Cluster 1 (Fig. 2C,D), Cluster 2 (Fig. 2G,H) exhibited a marked inflammatory exudate in the alveolar lumen, suggesting that the extent of consolidation was correlated with the intensity of pathological inflammation.

The clinical and radiological characteristics of the clusters are summarised in Table 2. The median time from symptom onset or initial visit to the SLB was similar between the groups, with no significant differences in sex or background diseases. Cluster 2 showed significantly reduced forced vital capacity (FVC) and forced expiratory volume in one second (FEV_1_), along with more pronounced hypoxia compared to Cluster 1. Serum Krebs von den Lungen-6 (KL-6) and erythrocyte sedimentation rate (ESR) were also elevated in Cluster 2. The parameter scores for each pathological finding in clusters 1 and 2 are also presented in Table 2. While there was no significant difference between the two groups in the extent of pathological fibrosis, as represented by fibrosis and atelectasis, inflammatory changes, including inflammatory cell infiltration and organizing pneumonia (OP), were markedly more pronounced in cluster 2. Notably, despite the absence of differences in clinical diagnoses between the two groups, features such as bronchiolitis, lymphoid follicle, plasma cell infiltration, and pleural involvements were more prominent in cluster 2.

**Table 2:** Comparison analysis of clinical, laboratory, and physiological data between cluster 1 and 2.

	Cluster1	Cluster2	p value
No. of cases	20	18	
Gender (male)	5 (25.0%)	9 (50.0%)	0.11
Never smoker	10 (50.0%)	7 (38.9%)	0.49
Days from first visit to SLB (days)	36.5 (18.8–74.5)	35 (13.0–45.5)	0.23
Days from symptom recognition to SLB (days)	85 (31.5–145.3)	106 (36.5–209.5)	0.36
Diagnosis			
IPAF	5 (25.0%)	4 (22.2%)	0.84
PM/DM	7 (35.0%)	9 (50.0%)	0.34
Other CTD	5 (25.0%)	1 (5.6%)	0.11
Pulmonary function test			
% FVC (%)	87.0 ± 16.4	71.3 ± 13.7	< 0.001*
%FEV_1_ (%)	84.8 ± 19.0	68.6 ± 15.7	< 0.001*
% DL_CO_ (%)	71.8 ± 18.0	64.5 ± 11.3	0.13
Blood test			
LDH (U/L)	215.5 (206.8–266)	251 (226–298.8)	0.23
KL-6 (U/mL)	1109 (874.8–1619.3)	2101 (1141–2922)	0.047*
CRP (mg/dL)	0.24 (0.11–0.72)	0.44 (0.11–0.76)	0.98
ESR (mm/h)	24.0 (18.0–34.0)	38 (23.8–51.0)	0.046*
pO_2_ (mmHg)	81.7 ± 9.1	75.7 ± 18.7	0.04*
Pathological evaluation (lower lobes)			
Alveolar epithelial injury	0 (0–1)	1 (0.25–2)	0.023*
Bronchiolitis	1 (0.75–1)	1 (1–1)	0.031*
Interstitial cellular infiltration	1 (1–1)	3 (2.25–3)	< 0.001*
Collapse	1 (0–1)	1 (0–2)	0.48
Fibrosis	2 (1–2)	1.5 (1–2)	0.47
Lymphoid follicle	0 (0–0)	1 (1–2)	< 0.001*
Mural incorporation fibrosis	1 (1–2)	2 (1.25–2)	0.07
Organising pneumonia	1 (0.75–1)	2 (2–2)	< 0.001*
Plasma cell infiltration	1 (1–1)	2 (1–2)	0.004*
Pleural fibrosis	0 (0–1)	1 (0.25–1)	0.004*
Pleuritis	0 (0–0)	0 (0–0.75)	0.03*
Traction bronchiectasis	1 (1–1)	1 (1–1)	0.14
Type II pneumocyte hyperplasia	1 (1–2)	1 (1–2)	0.43
Quantitative CT analysis (% lower lobes)			
Consolidation	2.46 (1.26–5.19)	4.66 (2.82–7.47)	0.037*
Consolidation with fibrosis	0.17 (0.05–0.39)	0.19 (0.04–0.34)	0.45
Emphysema	0.03 (0.00–0.22)	0.02 (0–0.04)	0.14
Fibrosis	0.09 (0.03–0.14)	0.09 (0.02–0.32)	0.37
Honeycomb	0 (0–0.002)	0.003 (0- 0.009)	0.073
GGO	7.48 (3.82–12.85)	8.68 (6.03–11.16)	0.34
Reticulation	4.04 (1.66–5.22)	4.80 (2.13–5.94)	0.25
Traction bronchiectasis	0.24 (0.15–0.54)	0.50 (0.29–0.83)	0.043*
Initial treatment			
OCS only	1 (5%)	2 (11.8%)	0.33
OCS + immunosuppressive	4 (16%)	5 (29.4%)	0.26
Pulse steroid therapy	10(50%)	9(52.9%)	0.21
At least 3-months follow-up	20 (100%)	16 (88.9%)	
Mortality	0 (0%)	2 (11.1%)	0.13
Number of acute exacerbation events	0 (0%)	0 (0%)	1
Frequency of PPF during follow-up	8/20 (40%)	12/16 (75%)	0.036*

*CTD* connective tissue disease, *DL*_*CO*_ diffusing capacity of the lungs for carbon monoxide, ESR erythrocyte sedimentation rate, *FEV*_1_ forced expiratory volume in one second, *FVC* forced vital capacity, *KL-6* Krebs von den Lungen-6, *OCS* oral corticosteroid, *PM/DM* polymyositis/dermatomyositis, *PPF* progressive pulmonary fibrosis, *SLB* surgical lung biopsy, *indicates p-value < 0.05.

Quantitative CT analysis of the lower lobes was performed to further evaluate radiological features (Table 2). Differences in the intensity of the HRCT findings between clusters were assessed by comparing the areas occupied by the radiological features. The analysis showed that the extent of consolidation and traction bronchiectasis was significantly greater in Cluster 2 than in Cluster 1.

No significant association was observed with the initial treatment regimen. However, the incidence of progressive pulmonary fibrosis (PPF)^17^ was notably higher in Cluster 2 than Cluster 1. Throughout the observation period, there were no cases of acute exacerbations. Mortality rates were similar, with no deaths in Cluster 1 and two in Cluster 2 during follow-up.

### Treatment response

**Fig. 3:**
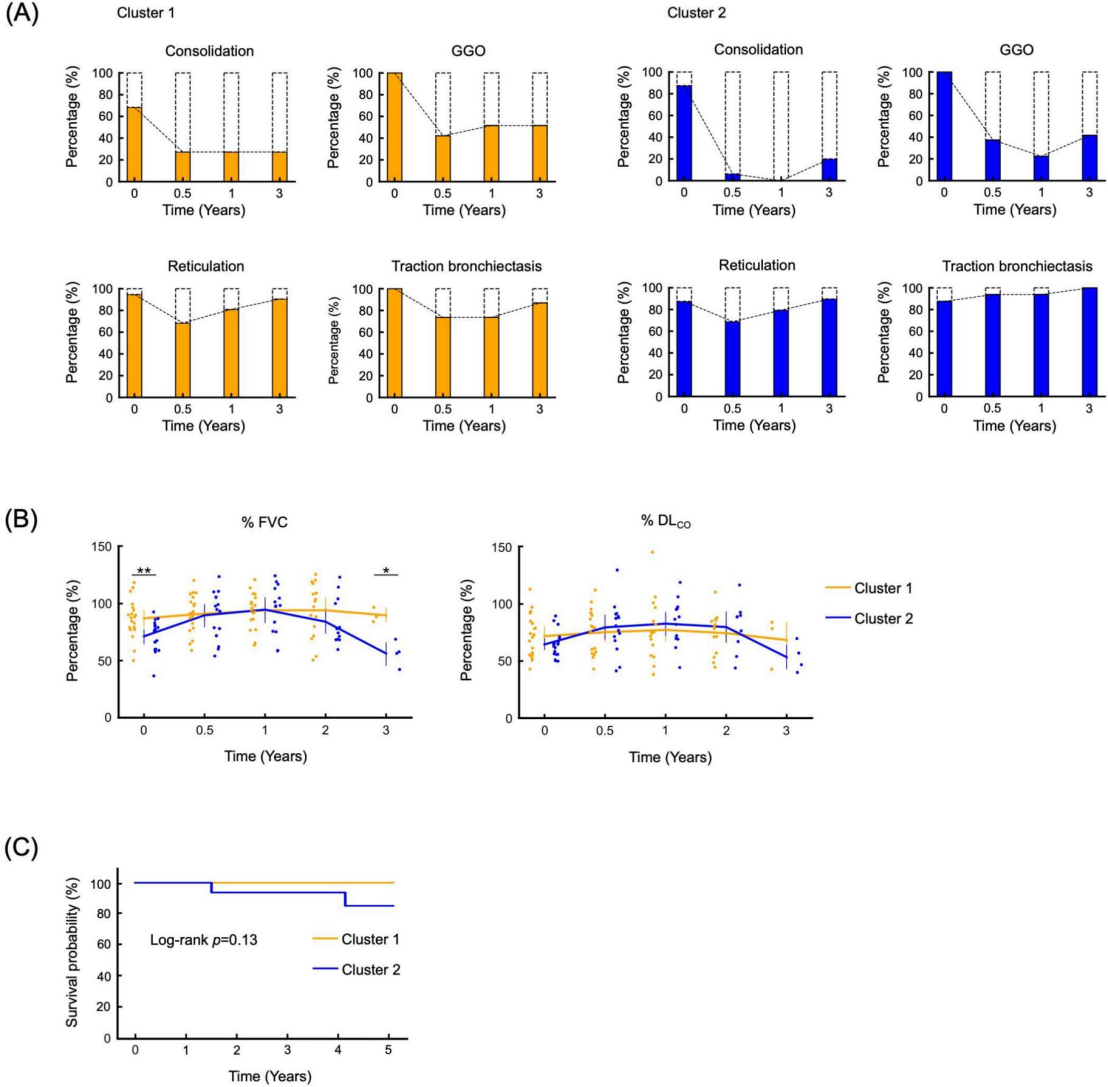
Changes of the intensity of high-resolution computed tomography findings and pulmonary function parameters during follow-up. (**A**) Changes of the intensity of each high-resolution computed tomography (HRCT) finding during follow-up periods. Coloured areas in the bar graph represent frequency of HRCT findings in each cluster at their initial scans and whether they were “worsened or unchanged” during follow-up periods. Proportions of broken line areas represent patient absence at their initial evaluation and whether their findings “improved or disappeared” during follow-up periods. Consolidation was the most sensitive lesion to treatment while reticulations and traction bronchiectasis progressed throughout the observation periods. (**B**) Time course changes of pulmonary function test parameters. Each dot represented data point for each case (orange, Cluster 1; blue, Cluster 2). %FVC changes were observed in clusters 1 and 2. Dramatic improvement after initial treatment was observed in Cluster 2; however, Cluster 2 showed marked re-worsening. Compared to %FVC, changes and difference in percentage of predicted diffusing capacity for carbon monoxide between clusters were modest. (**C**) Kaplan–Meier survival curves for Cluster 1 and Cluster 2. Each curve represents Cluster 1 and Cluster 2, respectively (orange, Cluster 1; blue, Cluster 2). %DLCO, percentage of predicted diffusing capacity of the lungs for carbon monoxide; %FVC, percentage of predicted forced vital capacity; GGO, ground-glass opacity.

Follow-up HRCT images were obtained from 35 patients (19 in Cluster 1 and 16 in Cluster 2). Three patients were transferred to other hospital before follow-up examinations were conducted. Figure 3A shows the changes in the treatment resistance ratio of each HRCT finding during follow-up. Consolidation and GGO were mostly resolved by the initial treatment. Resolution of consolidation was observed in all cases in Cluster 2 within a year, whereas five patients in Cluster 1 (26.3%) did not show improvement in consolidation during follow-up. Both Cluster 1 and Cluster 2 exhibited minimal improvement in reticulation and traction bronchiectasis. Notably, in Cluster 2, there was no observed improvement in traction bronchiectasis, indicating radiological progression.

The time courses of pulmonary function tests were also assessed in this study (Fig. 3B). Although percentage of predicted forced vital capacity (%FVC) was significantly lower in Cluster 2 than in Cluster 1 at baseline, a remarkable improvement was observed in Cluster 2. Moreover, the %FVC values at 1-year follow-up were almost comparable between clusters 1 and 2. This trend was compatible with the radiological improvement. While the improvement in %FVC was limited in Cluster 1, %FVC in Cluster 1 remained relatively stable during the observation period. In contrast, a significant re-worsening of %FVC was observed only in Cluster 2. All patients who experienced a decline in FVC were receiving oral tacrolimus in addition to prednisone at a maintenance dose of 5–10 mg per day. In all cases, dose reduction of these medications was not performed within the three months preceding the detection of FVC deterioration. While the change in percentage of predicted diffusing capacity for carbon monoxide over the follow-up period was mild compared to the change in %FVC, a similar trend was observed for both pulmonary function test parameters. Acute exacerbation events were not observed in either group during the period. Also, no significant difference in survival rates was observed between the two groups (Fig. 3C).

**Fig. 4:**
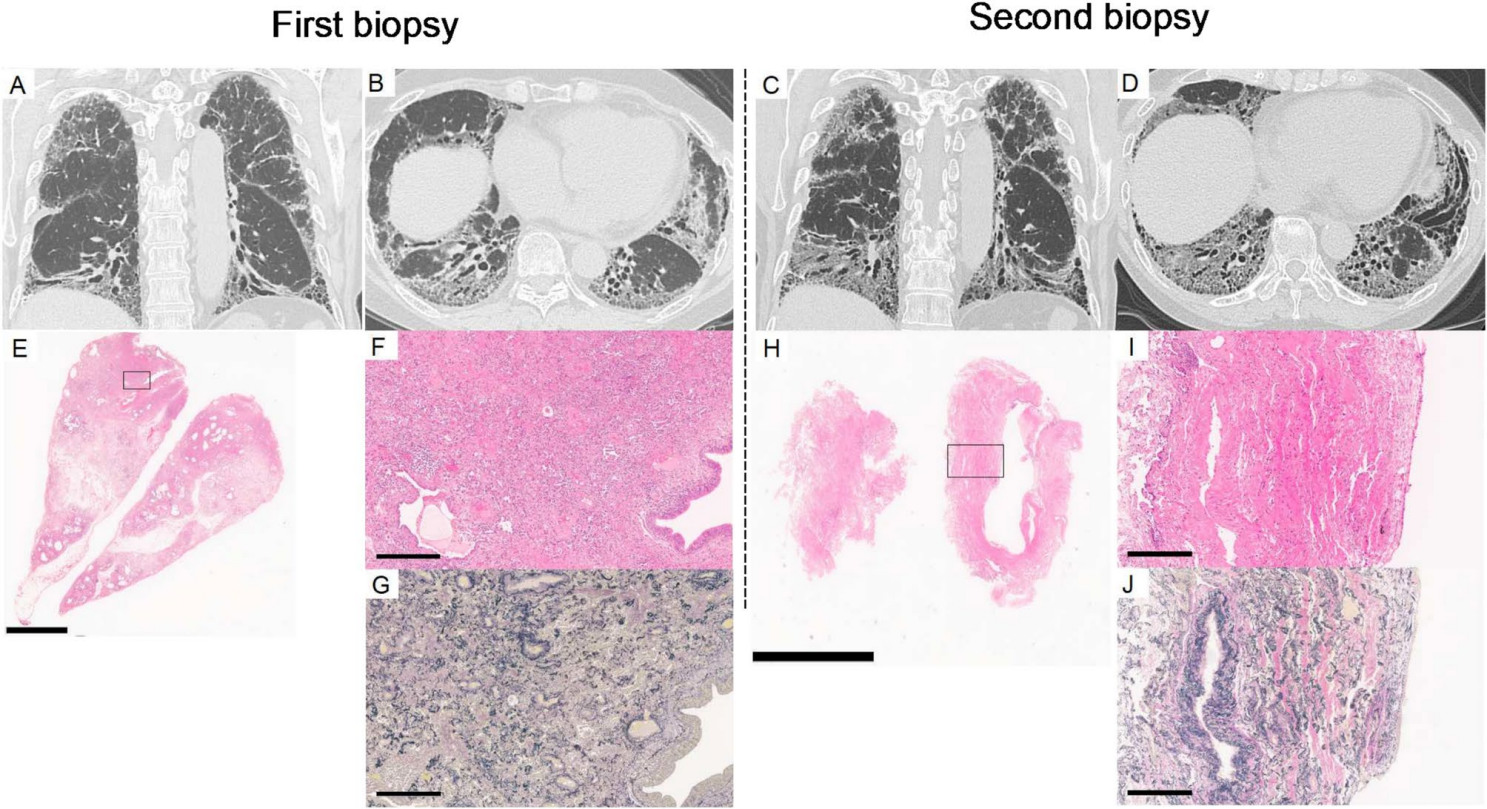
Representative radiological and pathological images of a Cluster 2 case with progressive pulmonary fibrosis. Representative high-resolution computed tomography (HRCT) images of a case in Cluster 2 that developed progressive pulmonary fibrosis (PPF), and pathological images of lung biopsy specimens collected by surgical lung biopsy (SLB) (first biopsy) and transbronchial lung cryobiopsy (TBLC) (second biopsy) were shown. Coronal (**A**) and axial (**B**) views at the initial visit, and coronal (**C**) and axial (**D**) sections from a 3-year follow-up HRCT taken just before transbronchial lung cryobiopsy (TBLC) are shown. Compared to HRCT images at first visit, progression of reticulation, traction bronchiectasis and ground-glass opacity (GGO) were observed at 3-year follow-up. A haematoxylin–eosin (HE)-stained overview (**E**), along with magnified HE-stained (**F**) and Elastica van Gieson (EVG)-stained images (**G**) of the specimen obtained from SLB, are shown (The scale bars represent 4 mm, 300 μm, and 300 μm, respectively). Similarly, HE-stained overview (**H**) and HE- (**I**) and EVG-stained (**J**) magnified images of the TBLC specimens are shown (The scale bars indicate 4 mm, 300 μm, and 300 μm, respectively). Compared with the SLB specimens which presented diffuse inflammatory cellular infiltration with fibrosis, the TBLC specimens showed a marked increase in fibrosis with collapsed airspace and a significant decrease in inflammatory cellular infiltration.

A representative case classified as Cluster 2 that underwent a secondary lung biopsy during the follow-up period is demonstrated in Fig. 4. The patient exhibited progressive worsening of ILD despite combination therapy with oral steroids and tacrolimus and subsequently received transbronchial lung cryobiopsy (TBLC). Compared to the initial study (Fig. 4A,B), three-year follow-up CT performed immediately before TBLC demonstrated progression of reticulation, traction bronchiectasis, and GGO, while consolidation was partially resolved (Fig. 4C,D). Lung tissue samples collected by SLB showed diffuse inflammatory cellular infiltration with fibrosis and architectural destruction (Fig. 4E–G). In contrast, TBLC specimens stained with HE and EVG demonstrated a significant decrease in inflammatory cellular infiltration and a marked increase in collagen fibre regions with collapsed airspaces (Fig. 4H–J). Taken together, our results demonstrated that Cluster 2 was predisposed to PPF despite partial improvement in inflammatory changes.

### ROC curve analyses for cluster differentiation

**Fig. 5:**
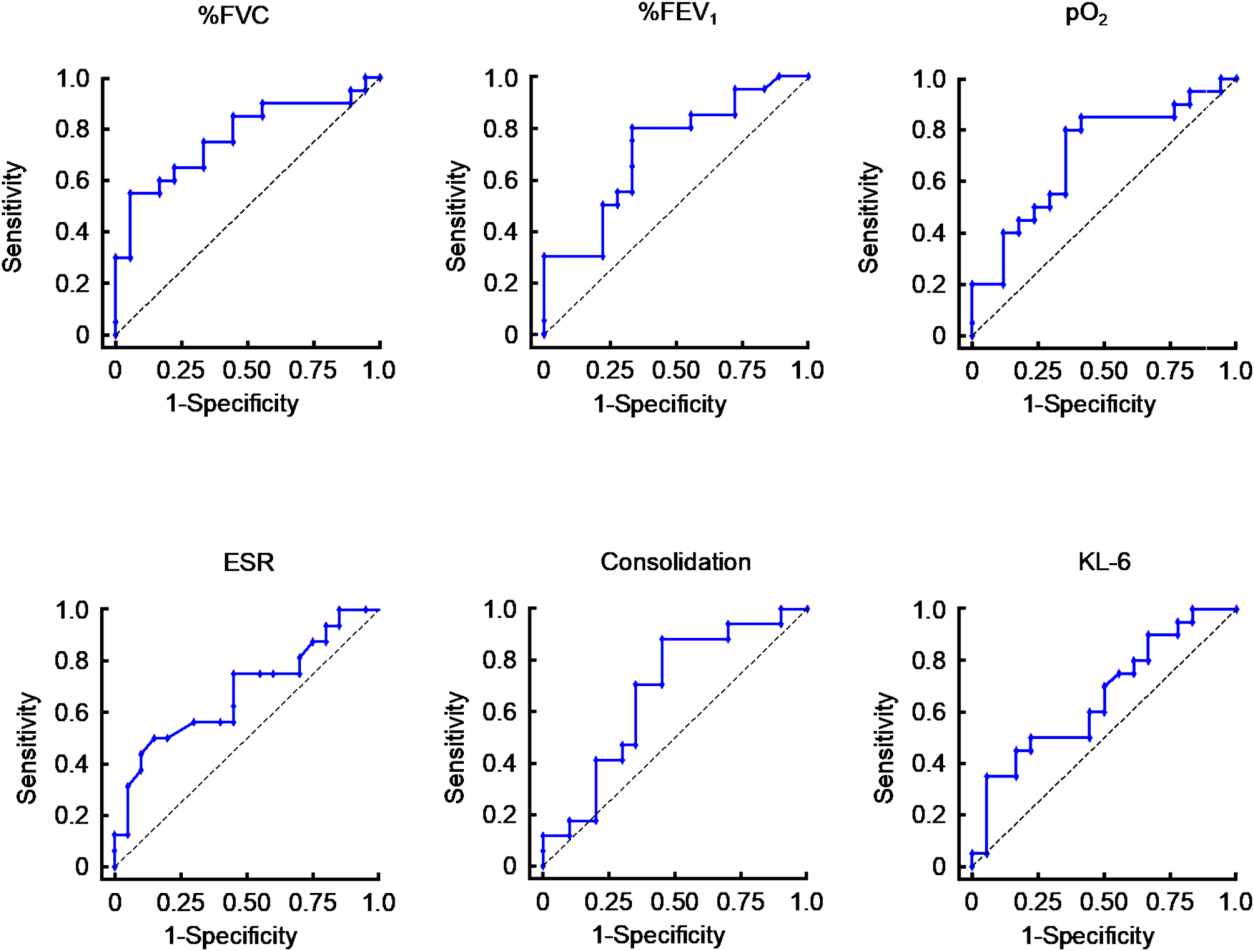
Receiver operating characteristic curve analysis for distinguishing clusters. Receiver operating characteristic (ROC) curve analysis of percentage of predicted forced vital capacity (%FVC), percentage of predicted forced expiratory volume in one second (%FEV_1_), arterial partial pressure of oxygen (Pa_O₂_), erythrocyte sedimentation rate (ESR), extent of consolidation in the lower lobes, and Krebs von den Lungen-6 (KL-6) were shown. These ROC curves provided the diagnostic utility of clinical and radiological evaluation in differentiating clusters 1 and 2. ESR, erythrocyte sedimentation rate; %FEV_1_, percentage of predicted forced expiratory volume in one second; %FVC, percentage of predicted forced vital capacity; KL-6, Krebs von den Lungen-6; Pa_O₂_, arterial partial pressure of oxygen.

Owing to the disease severity and marked progression during follow-up, early detection of Cluster 2 was imperative. ROC curve analysis was performed for clinical and imaging findings that showed statistically significant differences between the groups. Parameters with an AUC of 0.6 or higher were considered to have detection capability and are presented in Fig. 5. The key parameters for detection included %FVC, %FEV_1_, arterial partial pressure of oxygen (Pa_O2_), ESR, extent of consolidation in the lower lobes, and serum KL-6 levels. The following cutoff values were used for detection in Cluster 2: %FVC, 87.6% (sensitivity, 55.0%; specificity, 94.4%; AUC, 0.77 ± 0.15); %FEV_1_, 75.8% (sensitivity, 80.0%; specificity, 66.7%; AUC, 0.72 ± 0.17); Pa_O₂_, 77.0 Torr (sensitivity, 80.0%; specificity, 65.0%; AUC, 0.71 ± 0.18); ESR, 42 mm/hr (sensitivity, 50.0%; specificity, 85.0%; AUC, 0.68 ± 0.18);extent of consolidation in lower lobes, 2.62% (sensitivity, 88.2%; specificity, 55.0%; AUC, 0.67 ± 0.18); serum KL-6 levels, 0.2502 U/mL (sensitivity, 65.0%; specificity, 94.4%; AUC, 0.66 ± 0.18).

## Discussion

In this study, we demonstrated the pathological heterogeneity of NSIP/OP overlap and its association with radiological findings and clinical courses. According to the results of the two-dimensional PCA, the first principal component (x-axis) was associated with inflammatory changes, including inflammatory cellular infiltration, OP, and plasma cell infiltration. In contrast, the second principal component (y-axis) was linked to fibrotic features, such as fibrosis and traction bronchiectasis. Consequently, Cluster 2, identified as the first principal component high cluster, was characterised by intense inflammatory changes with fibrosis, whereas Cluster 1 exhibited fibrotic changes with mild inflammation. Representative pathological images corroborated these findings, showing that Cluster 2 had a significant extent of OP and marked inflammatory cellular infiltration with fibrosis, whereas Cluster 1 displayed modest inflammatory changes. Based on these findings, Cluster 2 could be considered as cellular NSIP/OP group, and Cluster 1 as fibrotic NSIP/OP group. There was a possibility that the observed differences between the two clusters could be attributed to differences in the time course; however, both the number of days from the first visit to SLB and the number of days from symptom recognition to SLB were almost comparable between the clusters. Thus, we conclude that each cluster was a distinct phenotype of NSIP/OP overlap.

While background diseases and comorbidities did not differ between the clusters, Cluster 2 was associated with more severe ILD and an increased risk of PPF. Patients in Cluster 2 exhibited higher KL-6 levels, indicating a potential link between elevated KL-6 levels and progressive ILD. Elevated KL-6 levels have been previously associated with higher mortality and poorer outcomes in patients with progressive fibrosing ILD and polymyositis/dermatomyositis-associated ILD^27–30^. Additionally, previous studies have reported that serum C-reactive protein (CRP) levels correlate with mortality^30,31^. Although our study found no significant differences in CRP levels between clusters, ESR levels were significantly higher in Cluster 2 than in Cluster 1. ESR levels have also been shown to correlate with increased ILD frequency and mortality in patients with dermatomyositis^32^. Taken together, these findings suggest that ESR may be more sensitive than CRP for detecting severe ILD, particularly in the context of NSIP/OP overlap. Previous studies have suggested that ferritin levels serve as a useful prognostic marker in myositis-associated interstitial lung disease. While ferritin may also be a valuable indicator in the present study, its evaluation prior to treatment was limited to a small number of cases, preventing further analysis.

Quantitative CT analysis showed that the extent of consolidation was significantly higher in Cluster 2 than in Cluster 1. Furthermore, follow-up evaluations revealed that in most cases within Cluster 2, consolidation improved within six months. These results suggest a potential correlation between consolidation and inflammatory changes that are responsive to immunomodulatory therapy.

NSIP/OP overlap is often associated with anti-ARS antibodies^2,3,9,11,33–35^. Nevertheless, in our study, over half of the cases were found to be anti-ARS antibody-negative, indicating a more diverse background. Indeed, 13% of participants were previously diagnosed with systemic sclerosis. In addition, NSIP/OP overlap exhibited the highest survival rate among NSIP overlap with other pathological patterns, suggesting that OP is one of the factors associated with better prognosis^16,17^. However, although several cases of NSIP/OP overlap resolved successfully with treatment, most showed progression during follow-up^15,37^, suggesting it should be considered a progressive disease.

In our study, the majority of patients with NSIP/OP overlap demonstrated disease progression during three-year follow-up. While consolidation and GGO responded well to treatment, the improvement of fibrotic features, including reticulation, was limited, and most cases even exhibited progression of traction bronchiectasis within 3-year follow-up. In other words, radiological fibrotic changes in NSIP/OP overlap progressed during the clinical course.

Progressive pulmonary function deterioration, particularly in Cluster 2 patients, mirrored the CT changes observed during follow-up. The initial improvement in pulmonary function due to resolution of inflammation was followed by a significant decline in FVC in Cluster 2, driven by progressive fibrosis. This finding was consistent with the higher incidence of PPF observed in this cluster.

Notably, while the initial therapeutic strategies were almost comparable between clusters, significant changes in the clinical courses eventually emerged. Although OP, initially deemed a favourable prognostic factor, was more prominent in Cluster 2, our results indicated significant progression in this cluster. This discrepancy may be attributed to the accompanying alveolar epithelial injury, which could promote fibrosis during lung injury repair. It is also possible that the increased serum KL-6 levels observed in Cluster 2 were due to alveolar epithelial injury.

Follow-up biopsy results from patients in Cluster 2 also showed progressive fibrosis and resolution of inflammatory changes with conventional anti-inflammatory therapy. Therefore, re-examination of lung pathology in progressive cases is crucial for evaluating the potential overuse of immunosuppressive drugs such as corticosteroids. While current guidelines on TBLC have made no reference to the usability of TBLC for the follow-up of patients with ILD^38^, TBLC could be a promising tool for the reassessment of therapeutic strategies in patients with ILD.

ROC curve analysis indicated that lower pulmonary function, hypoxia, increased intensity of consolidation, and elevated ESR level were the effective markers for predicting Cluster 2. This result was rational because reduced pulmonary function, elevated inflammatory marker, and the extent of consolidation might be positively associated with lung inflammation and alveolar epithelial damage. Consequently, we concluded that the severity of lung impairment at the initial visit could influence the long-term clinical course and prognosis.

The optimal therapeutic strategy for patients with NSIP/OP remains controversial. As most cases in our study showed improvement with initial treatment, the treatment responsiveness of NSIP/OP was considered to be favourable. Nevertheless, a decline in FVC was observed over long-term follow-up, and some cases progressed to PPF. To facilitate the early detection of progression, comprehensive monitoring with periodic pulmonary function tests every 3 to 6 months, as recommended by the American College of Rheumatology, is considered necessary as a monitoring test^39^. As suggested by the results of the INBUILD study and the concept of PPF^19,40^, antifibrotic therapy might hold potential benefits for the long-term management of NSIP/OP. Additionally, the EVER-ILD study demonstrated that the addition of rituximab (RTX) to mycophenolate mofetil (MMF) treatment significantly controlled the decline in FVC in patients with CTD-ILD and IIP with an NSIP pattern^41^. The RECITAL study also suggested that RTX should be considered as an alternative to cyclophosphamide for intravenous therapy in individuals with CTD-ILD^42^. The latest ACR guideline recommend mycophenolate, RTX, cyclophosphamide, and nintedanib for patients with systemic autoimmune rheumatic disease-associated ILD that progresses despite initial treatment^43^. Also, RTX is preferred as the first-line treatment for myositis-associated ILD, which is prone to presenting with an NSIP/OP pattern. In this study, not all cases met the criteria for CTD-ILD; however, it focused on a cohort of interstitial lung diseases with an NSIP component. While antifibrotic therapy may be considered in accordance with PPF management, based on these previous reports, the escalation of anti-inflammatory treatment, including RTX, prior to antifibrotic therapy may offer potential benefits in improving or stabilizing FVC.

This study had several limitations owing to its retrospective nature. First, this study was a single-centre study with limited ethnic diversity. Multivariate analysis was not feasible due to its sample size. Second, while there were no differences in treatment strategy between clusters, the dose and duration of immunomodulatory therapy was not standardized. Third, owing to the high survival rate, evaluation of prognosis between clusters was limited. Lastly, this cohort inherently excluded cases in which patients either refused surgical biopsy or were too frail to undergo the procedure, resulting in a potential intrinsic bias that could not be entirely ruled out.

Despite these limitations, this study represents a comprehensive investigation summarising the inherent heterogeneity of NSIP/OP overlap and provides new insights that could guide long-term treatment strategies by utilizing three prognostic factors: hypoxia, greater extent of consolidation, and lowered FVC at the initial consultation. Nevertheless, future studies with larger sample sizes, diverse patient backgrounds, standardized treatment strategies, and thorough multivariate analyses are warranted to further expand our understanding of NSIP/OP overlap and its prognosis.

## Conclusion

In this study, pathological heterogeneity in NSIP/OP overlap was identified using clustering analysis, which was associated not only with the severity of ILD at the initial visit but also with long-term clinical courses. While initial therapeutic responses were observed in both clusters, differences in disease progression during follow-up and the frequency of developing PPF were noted. Our study suggests that FVC, FEV_1_, Pa_O₂_ the extent of consolidation in the lower lobes, and serum KL-6 level serve as markers or differentiating clusters in NSIP/OP overlap that could potentially affect long-term prognosis.

Given that NSIP/OP overlap is heterogeneous and can follow a progressive course, careful long-term observation and reassessment of pulmonary pathology using diagnostic methods such as TBLC are crucial for re-evaluating treatment strategies.

## Data Availability

The datasets used and/or analysed during the current study are available from the corresponding author on reasonable request.

## Abbreviations

%FVC: Percentage of predicted forced vital capacity
ARS: Aminoacyl-tRNA synthetase
AUC: Area under curve
CRP: C-reactive protein
CT: Computed tomography
ESR: Erythrocyte sedimentation rate
EVG: Elastica van Gieson
FVC: Forced vital capacity
GGO: Ground-glass opacity
HE: Haematoxylin–eosin
HRCT: High-resolution computed tomography
IIP: Idiopathic interstitial pneumonia
ILD: Interstitial lung disease
KL-6: Krebs von den Lungen-6
MDD: Multidisciplinary discussion
NSIP: Nonspecific interstitial pneumonia
NSIP/OP overlap: Nonspecific interstitial pneumonia with organising pneumonia overlap
OP: Organising pneumonia
PCA: Principal component analysis
Pa_O2_: Arterial partial pressure of oxygen
PPF: Progressive pulmonary fibrosis
ROC: Receiver operating characteristic
SLB: Surgical lung biopsy
UIP: Usual interstitial pneumonia
